# Extra-articular deformity correction using Taylor spatial frame prior to total knee arthroplasty

**DOI:** 10.1007/s11751-018-0310-5

**Published:** 2018-03-20

**Authors:** Gautam J. K. Tawari, Rajan Maheshwari, Sanjeev S. Madan

**Affiliations:** Department of Orthopaedics, Doncaster Royal Infirmary, Armthorpe Road, Doncaster, DN2 5LT UK

**Keywords:** Tibial deformity, Tibial non-union, Total knee arthroplasty, Staged surgery, Osteoarthritis

## Abstract

A good long-term outcome following a total knee arthroplasty relies on restoration of the mechanical axis and effective soft tissue balancing of the prosthetic knee. Arthroplasty surgery in patients with secondary osteoarthritis of the knee with an extra-articular tibial deformity is a complex and challenging procedure. The correction of mal-alignment of the mechanical axis is associated with unpredictable result and with higher revision rates. Single-staged deformity correction and replacement surgery often result in the use of constraint implants. We describe our experience with staged correction of deformity using a Taylor Spatial Frame (TSF) followed by total knee arthroplasty in these patients and highlight the advantage of staged approach. The use of TSF fixator for deformity correction prior to a primary total knee arthroplasty has not been described in the literature. We describe three cases of secondary osteoarthritis of the knee associated with multiplanar tibial deformity treated effectively with a total knee arthroplasty following deformity correction and union using a TSF. All patients had an improved Knee Society score and Oxford Knee score postoperatively and were satisfied with their replacement outcome. Staged deformity correction followed by arthroplasty allows the use of standard primary arthroplasty implants with predicable results and flexible aftercare. This approach may also provide significant improvement of patient symptoms following correction of deformity resulting in deferment of the arthroplasty surgery.

## Introduction

A good long-term outcome following a total knee arthroplasty relies on restoration of the mechanical axis and effective soft tissue balancing of the prosthetic knee [1]. Severe osteoarthritis of the knee associated with peri-articular deformity can be effectively addressed by appropriate bony resections during the surgical implantation of a prosthetic total knee [2]. Secondary osteoarthritis of the knee with an extra-articular tibial deformity or a proximal tibial non-union presents a surgical challenge to obtain correction of alignment or achieve union with a total knee arthroplasty, without prior correction of the deformity. We describe our experience with secondary osteoarthritis of the knee associated with multiplanar tibial deformity in two cases and with stress fracture non-union in one case, treated effectively with a total knee arthroplasty following deformity correction and union using a Taylor spatial frame (TSF).

## Case 1

**Fig. 1:**
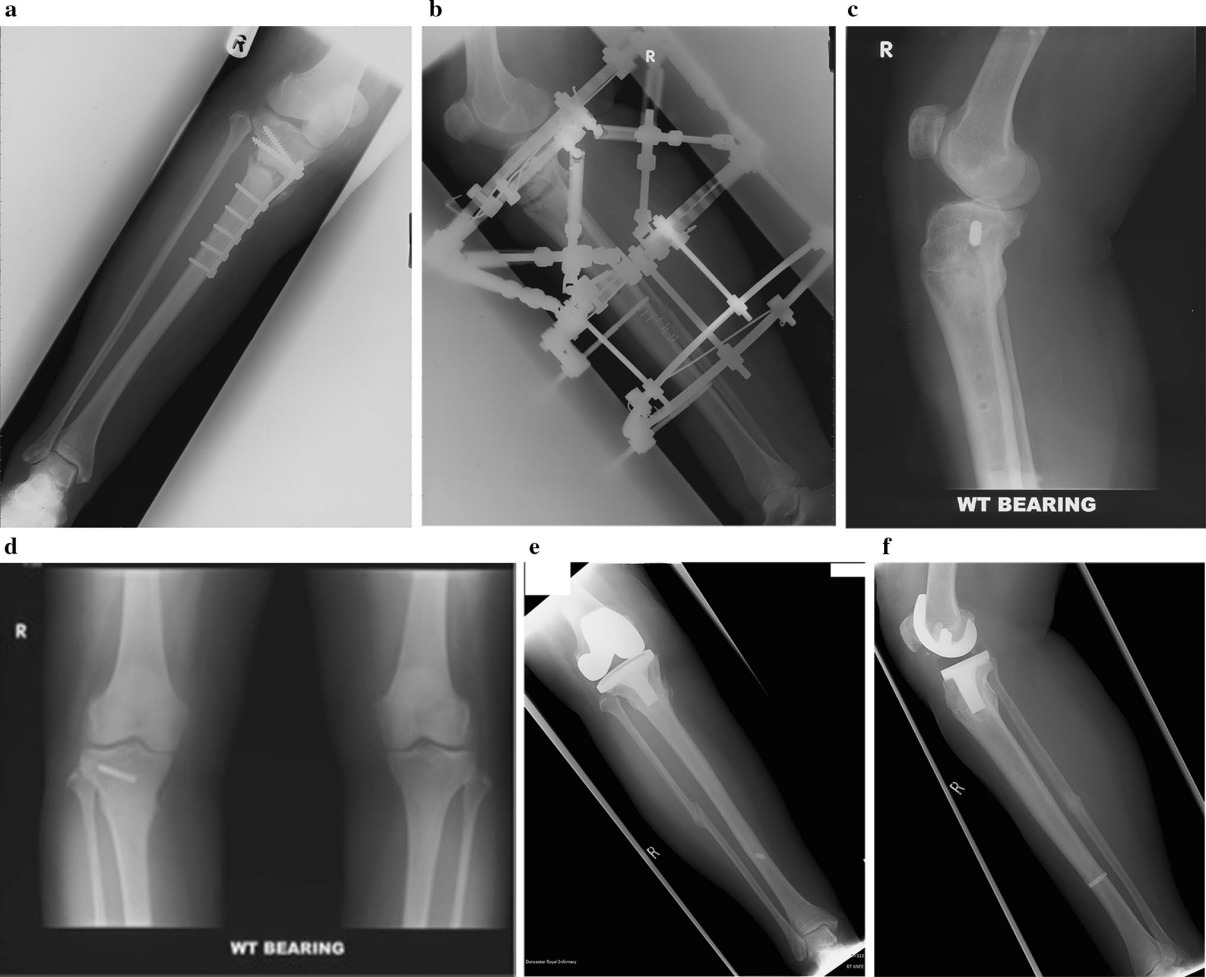
Case 1: **a** Radiograph showing established proximal tibial non-union following stress fracture, failed treatment of internal fixation and bone graft augmentation. **b** Radiograph showing frame fixation for treatment of proximal tibial non-union and deformity correction. **c**, **d** Orthogonal radiographs showing union of the stress fracture. **e**, **f** Orthogonal radiographs showing total knee replacement in situ performed as a staged procedure

A 63-year-old female presented with an established non-union of a proximal tibial stress fracture and secondary osteoarthritis of the knee. The stress fracture had failed to unite over a period of 2 years despite attempts at both conservative and surgical treatment with proximal tibia plating and bone graft augmentation. She had a residual 10 degrees of varus proximal tibial deformity and non-union with an Oxford knee score of 22 (Fig. 1).

She underwent correction of deformity and treatment of non-union using principles of distraction osteogenesis in a TSF fixator. The frame fixator was stabilised with two half pins and an olive wire for the proximal ring and three olive wires for the distal ring. The two rings were connected with six struts, and a computer-generated programme was used for correction. An uneventful deformity correction along with proximal tibial union was achieved at 42 weeks.

The lady then had a primary total knee replacement 18 months following the corrective surgery for symptomatic secondary knee osteoarthritis. At 5 years follow-up, she had a flexion range of 0–90 degrees in the knee with a Knee Society score of 89 (functional score of 90) and the Oxford knee score of 38.

## Case 2

**Fig. 2:**
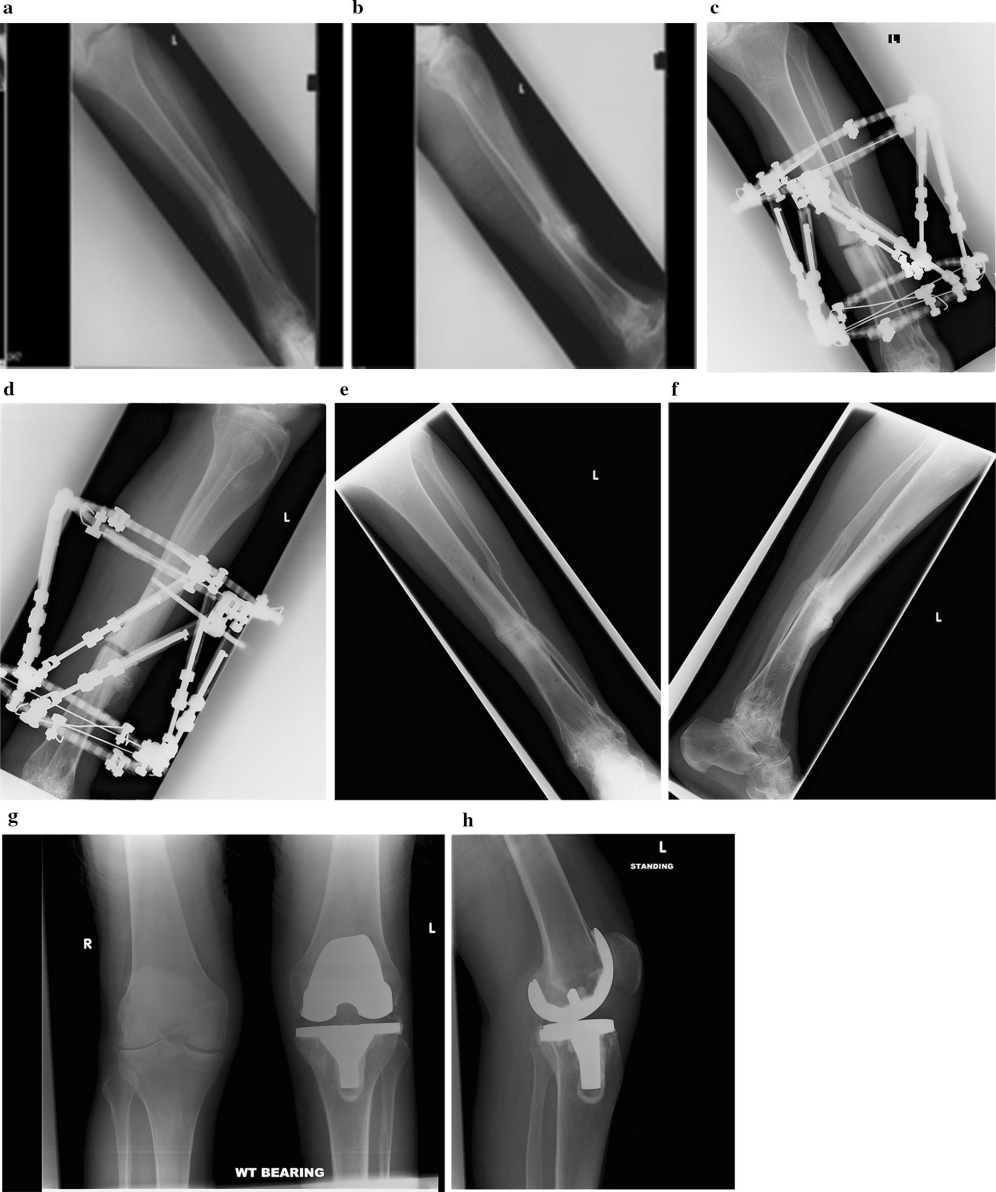
Case 2: **a**, **b** Orthogonal radiographs of tibial malunion deformity used for deformity correction planning. **c**, **d** Orthogonal radiographs showing Taylor spatial frame application with corrective tibial osteotomy away form the CORA. **e**, **f** Orthogonal radiograph showing realignment of the mechanical axis and healing of the osteotomy site with an established ankle arthrodesis. **g**, **h** Orthogonal radiographs showing total knee replacement in situ performed as a staged procedure

A 64-year-old male presented with a post-traumatic tibial deformity and secondary osteoarthritis of the knee. The predominant tibial deformity following the malunion was 10 mm shortening, 8 degrees of varus and 7 degrees of recurvatum and an Oxford knee score of 16. The planning for corrective surgery was further strained by the presence of an ipsilateral ankle arthrodesis in this patient (Fig. 2).

He underwent corrective osteotomy to allow adequate correction of the deformity and realignment of the tibial mechanical axis using a Taylor spatial frame. A corticotomy was performed at 90 mm proximal to the centre of rotation of angulation for the deformity (CORA). This resulted in 10 mm of posterior translation at the corticotomy site. The corticotomy was stabilised using two half pins and a wire for the proximal ring and three olive wires for the distal ring. The two rings were connected using six struts, and the deformity corrected using a computer-generated correction programme. The correction and union at corticotomy site were achieved at 38 weeks.

He subsequently underwent an uneventful primary total knee arthroplasty 24 months following the deformity correction for symptomatic secondary knee osteoarthritis. At 4 years follow-up, he had a flexion range of 0–100 degrees in the knee and a Knee Society score of 90 [functional component 90] and an Oxford knee score of 41.

## Case 3

**Fig. 3:**
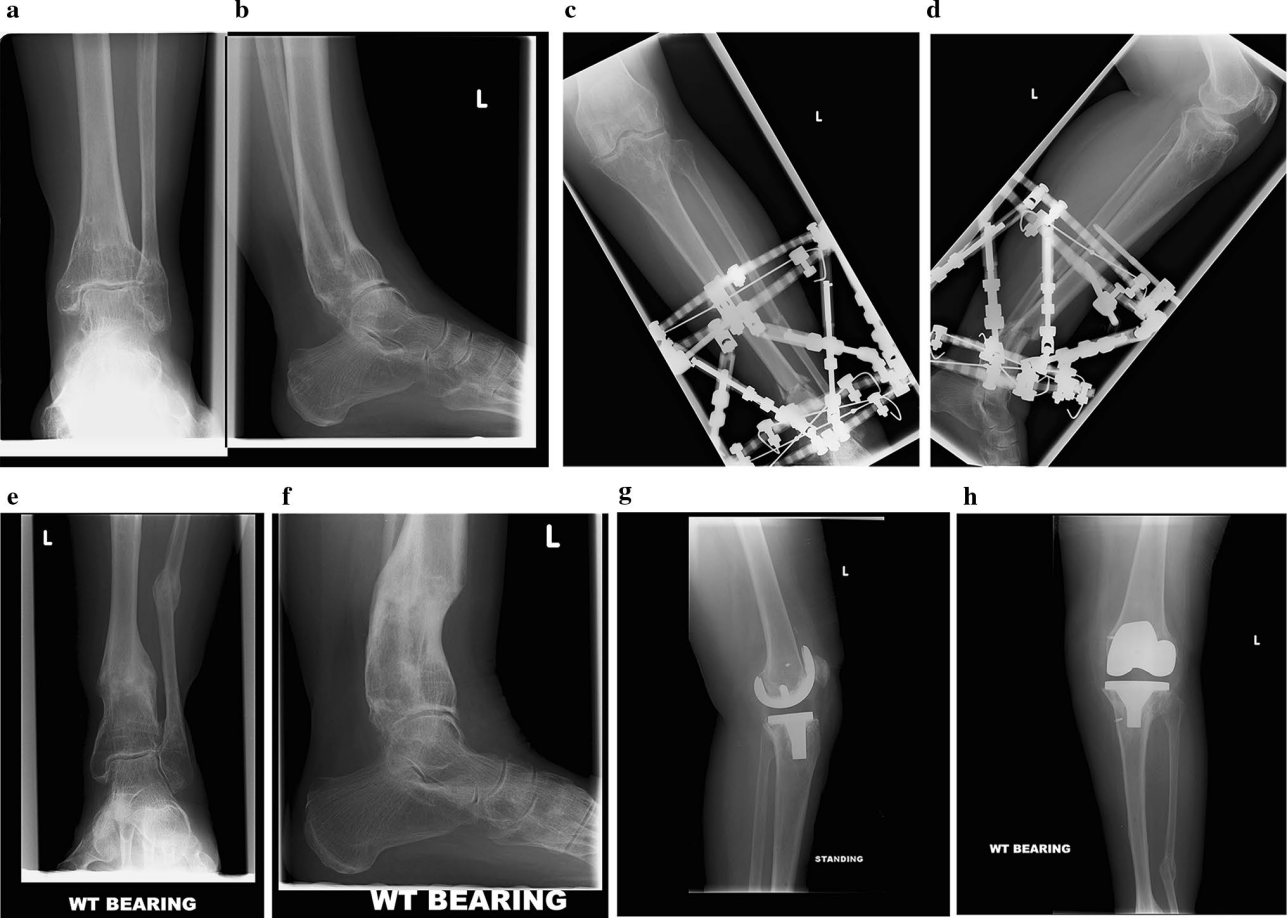
Case 3: **a**, **b** Orthogonal radiographs showing supramalleolar tibial deformity. **c**, **d** Orthogonal radiographs showing application of Taylor spatial frame and tibial osteotomy for correction of deformity. **e**, **f** Orthogonal radiographs showing realignment of mechanical axis with healing of the osteotomy site after removal of frame. **g**, **h** Orthogonal radiographs showing total knee replacement in situ performed as a staged procedure

A 63-year-old female presented with a tibial deformity and secondary osteoarthritis of the knee. The tibial deformity was multiplanar with a proximal tibial valgus of 12 degrees, external tibial torsion of 20 degrees and a distal tibial recurvatum of 30 degrees. The proximal tibial deformity was secondary to lateral tibial plateau fracture, whereas the supramalleolar ankle deformity was secondary to distal tibial malunion following a road traffic accident 20 years earlier. She had an Oxford knee score of 18 (Fig. 3).

She underwent correction of external tibial torsion (rotational deformity) and recurvatum at the distal tibial using a Taylor spatial frame with supramalleolar corticotomy. The corticotomy was stabilised using two half pins and an olive wire for the proximal ring and three olive wires for the distal ring. The two rings were connected using six struts and the deformity correction undertaken using a computer-generated programme. During the course of correction, this patient required a short course of oral antibiotics for pin site infection and also had a reoperation for readjustment of her frame. Correction of the deformity was achieved at 40 weeks with removal of the frame. She underwent a total knee replacement at 18 months following the deformity correction for symptomatic secondary osteoarthritis. The peri-articular valgus deformity of the knee was addressed with bony resection intraoperatively. She was also found to have an attenuated medial collateral ligament of the knee which required an intraoperative ligament reconstruction using Mitek^®^ bone anchors and semitendinosus graft. At 4 years follow-up, she had a flexion range of 0–90 degrees in the knee with a Knee Society score of 81 (functional score 75) and an Oxford knee score of 35.

## Discussion

Extra-articular deformity of the tibia associated with symptomatic secondary osteoarthritis of the knee requires a total knee arthroplasty. The presence of mal-alignment of the mechanical axis makes the intervention challenging and its result unpredictable with higher revision rates secondary to loosening of tibial component [3, 4]. The most common causes for extra-articular deformity around the knee are malunion, non-union and metabolic bone diseases like Paget’s, osteomalacia or rickets.

An extra-articular deformity with a total knee replacement of more than 10 degrees in coronal plane [Varus/Valgus] or more than 20 degrees in sagittal plane [Procurvatum/Recurvatum] requires a corrective osteotomy to obtain a satisfactory result. Intra-articular techniques using bone resections and soft tissue releases for significant peri-articular deformities result in complex ligament imbalances often then requiring ligamentous reinforcement, use of constraint implants or custom-designed prosthesis to obtain satisfactory soft tissue balance [5]. Correction of extra-articular deformity with the use of intra-articular techniques also results in such corrections to be away from the centre of rotation of angulation (CORA) of the deformity and can result in translation at the resection site and make the long-term outcome of the implant unpredictable.

Deformity correction and total knee replacement can be performed as a single-stage procedure or a two-stage procedure. Proponents of single-stage correction and arthroplasty allow extensive bone resection and often use a stemmed prosthesis to achieve stability at the osteotomy site. The literature reflects on such techniques with short-term outcomes, and regular long-term follow-up for these patients is routinely advocated. Distal femoral deformities are more amenable to such single-stage techniques as opposed to correction of tibial deformity [6–12].

The use of ring fixator with Ilizarov technique has been described for treatment of periprosthetic fractures and deformity correction prior to revision knee replacement. In our knowledge, the use of Taylor spatial frame fixator for deformity correction prior to a primary total knee arthroplasty has not been described in the literature. In our senior author’s experience, two earlier patients (not included in this description) with tibial deformity and concurrent knee osteoarthritis treated with initial deformity correction performed as a staged procedure experienced a significant respite from their osteoarthritic symptoms. These two patients continue to remain symptomatically well managed and are still awaiting the need of replacement surgery.

In our first patient, the proximal tibial non-union was secondary to a stress fracture. This had failed union at previous attempt involving internal fixation and bone graft augmentation. Proximal tibial non-union is rare and accounts for 3% of cases; failed internal fixation with bone graft augmentation of such non-union warrants the use of ring fixator to achieve union [13]. We felt it was important to achieve union prior to arthroplasty to obtain a predictable outcome. A single-stage stemmed approach with an underlying non-union would have been on course for failure, in turn limiting further revision options. Repeat use of internal fixation was deemed unsuitable due to previous incisions, soft tissue and bony devitalisation, short proximal bone segment with previous screw holes limiting the purchase of new hardware with compromised stability.

In both our second and third patients, the deformity was multiplanar and much distal to the knee joint. The use of internal fixation would have resulted in a period of immobilisation due to lack of weight bearing stability along with awkward incisional planes and risk of devitalisation of soft tissue and bony architecture jeopardising the final outcome of our total knee arthroplasty. The stability obtained with a TSF allowed early weight bearing and provided an ideal environment for new-bone formation and soft tissue healing following corrective osteotomies.

Our complications with this technique include one pin site infection successfully treated with a short course of oral antibiotics and one episode of frame readjustment under anaesthetic. We noted an average delay of 20 months between removal of the frame and total knee arthroplasty, our preference has been to allow approximately 12 months between procedures (removal of frame and total knee arthroplasty) to minimise our risk of infection and we have observed a symptomatic improvement in pain related to secondary arthritis following alignment of the mechanical axis. The longer duration of frame treatment in these cases reflects our protective attitude towards these patients due to the complex nature of their deformity and a foresight towards a subsequent second stage in terms of a total knee arthroplasty to be performed. All patients were extremely satisfied with the outcome of their arthroplasty with a mean Knee Society score of 86 (functional component 85) and a mean Oxford knee score of 38.

## Conclusion

In our experience, an extra-articular tibial deformity with secondary osteoarthritis of the knee joint requires correction of the deformity (mechanical axis realignment) prior to performing a primary total knee arthroplasty. A total knee arthroplasty can then be successfully performed using standard prosthesis and technique with a much predictable outcome. We have observed improvement in patient’s pain following the deformity correction allowing us an interval of 20 months between the two surgeries. We feel with the use of standard prosthetic implants for knee arthroplasty, the long-term outcome in these patients should be no different from a standard primary knee joint replacement and their follow-up appointments follow standard hospital protocol. We think these patients would not require follow-up aftercare like patients with revision surgery and stemmed implants; however, our experience is limited and the condition itself continues to be an uncommon problem.

## References

[1] Wolff AM, Hungerford DS, Pepe CL (1991). The effect of extraarticular varus and valgus deformity on total knee arthroplasty. Clin Orthop Relat Res.

[2] Lonner JH, Siliski JM, Lotke PA (2000). Simultaneous femoral osteotomy and total knee arthroplasty for treatment of osteoarthritis associated with severe extra-articular deformity. J Bone Joint Surg Am.

[3] Catonne YRJ, Delattre O (2001). TKR in patients with severe extra-articular deformity—a review of 30 cases. J Bone Joint Surg Br.

[4] Milner SA, Davis TR, Muir KR, Greenwood DC, Doherty M (2002). Long-term outcome after tibial shaft fracture: is malunion important?. J Bone Joint Surg Am.

[5] Papadopoulos EC, Parvizi J, Lai CH, Lewallen DG (2002). Total knee arthroplasty following prior distal femoral fracture. Knee.

[6] Anderson SP, Matthews LS, Kaufer H (1990). Treatment of juxtaarticular nonunion fractures at the knee with long-stem total knee arthroplasty. Clin Orthop Relat Res.

[7] Moskal JTMJ (2001). Simultaneous management of ipsilateral gonarthritis and ununited tibial stress fracture: combined total knee arthroplasty and internal fixation. J Arthroplasty.

[8] Sawant MR, Bendall SP, Kavanagh TG, Citron ND (1999). Nonunion of tibial stress fractures in patients with deformed arthritic knees. Treatment using modular total knee arthroplasty. J Bone Joint Surg Br.

[9] Wang JW, Wang CJ (2002). Total knee arthroplasty for arthritis of the knee with extra-articular deformity. J Bone Joint Surg Am.

[10] Karatosun V, Alekberov C (2001). The Ilizarov method in total knee arthroplasty with nonunion of the proximal tibia–a case report. Acta Orthop Scand.

[11] Simon RGBM (1999). Use of Ilizarov external fixation for a periprosthetic supracondylar femur fracture. J Arthroplasty.

[12] Tate RAG, Scotland TR (2008). Use of an ilizarov fixator for deformity correction prior to revision knee arthroplasty. Bull NYU Hosp Joint Diseases.

[13] Carpenter CA, Jupiter JB (1996). Blade plate reconstruction of metaphyseal nonunion of the tibia. Clin Orthop Relat Res.

